# Machine learning models powered by emergency medical services data enhance stroke triage in prehospital settings

**DOI:** 10.1038/s41598-026-37069-x

**Published:** 2026-02-03

**Authors:** Michael Saban, Grant Hiura, Paula de la Peña, Amy Wozniak, Daniel Heiferman, Oguz Akbilgic, Mark Cichon, Samie Tootooni

**Affiliations:** 1 Department of Health Informatics and Data Science, Loyola University Chicago, 2160 S. First Avenue, Maywood, IL 60153 USA; 2 Department of Computer Science, Loyola University Chicago, Chicago, IL USA; 3 Internal Medicine and Pediatrics, Duke University Health System, Durham, NC USA; 4 Marcella Niehoff School of Nursing, Loyola University Chicago, Maywood, IL USA; 5 Clinical Research Office, Loyola University Chicago, Maywood, IL USA; 6 Department of Neurological Surgery, Endeavor Health, Naperville, IL USA; 7 Department of Cardiology, Wake Forest School of Medicine, Winston-Salem, NC USA; 8 Department of Emergency Medicine, Loyola University Medical Center, Maywood, IL USA

**Keywords:** Stroke, Prehospital data, Emergency medical services, Artificial intelligence, Decision support, Emergency department, Stroke, Diagnostic markers, Computer science

## Abstract

Timely stroke diagnosis is essential for delivering life-saving treatments, yet current prehospital stroke assessment tools often lead to missed or delayed diagnoses. Emergency Medical Services (EMS) increasingly collect detailed data during transport, offering an opportunity to develop AI-based tools to support early stroke detection. In this retrospective study, we evaluated the availability and reliability of prehospital data compared to Emergency Department (ED) records. We tested the potential of machine learning to support EMS stroke triage. Our cohort included 4,333 patients across 8,221 ambulance encounters from 2015 to 2020 with stroke rate of 2.0% (64% severe strokes). Vital signs such as heart rate, respiratory rate, blood pressure, oxygen saturation, and GCS scores, recorded in over 88%, were generally higher than their ED counterparts. We trained and evaluated random forest, XGBoost, and sequential neural networks for detecting stroke and severe stroke. The XGBoost model performed best for stroke detection (ROC-AUC 0.843 [0.77–0.89], PR-AUC 0.293 [0.16–0.45]), while Random Forest performed best for severe strokes (ROC-AUC 0.826 [0.75–0.90], PR-AUC 0.186 [0.07–0.35]). Models were calibrated to improve reliability, and feature importance was assessed using SHAP to enhance interpretability. These findings highlight the promise of AI-based tools in improving prehospital stroke triage with real-time EMS data.

**Supplementary Information:**

The online version contains supplementary material available at 10.1038/s41598-026-37069-x.

## Introduction

Stroke, which affects over 795,000 individuals each year in the United States, is a leading cause of preventable disability^1^. More recent advances in stroke care have allowed for reductions in morbidity and mortality, however these interventions become less effective the longer the time from stroke onset to treatment takes^2,3^. Certain stroke subtypes, for example ischemic stroke with large vessel occlusion (LVO) and ruptured aneurysmal subarachnoid hemorrhage (SAH), require prompt intervention to prevent immediate mortality and promote favorable long-term outcomes and minimization of persisting disability. Early recognition of stroke is essential across the entire prehospital workflow.

As the first point of contact, 9-1-1 dispatch centers are critical in initiating stroke care by recognizing potential stroke symptoms and activating the appropriate EMS response. Accurate dispatcher recognition has been associated with faster triage, greater likelihood of admission to endovascular-capable centers, and higher treatment rates for stroke^4^. Importantly, dispatcher labeling of calls as suspected stroke can influence EMS personnel’s initial assessment and prioritization at the scene. Once dispatched, Emergency Medical Services (EMS) play a crucial role in ensuring timely care through rapid triage and transport to the nearest facility with appropriate treatment capabilities.

Routing decisions are guided by algorithms that prioritize minimizing delays while directing patients to high-capability centers^5,6^. However, diagnostic errors or limitations in the tools available to assess stroke severity lead to (1) unnecessary transport to distant specialized centers, causing treatment delays; or (2) initial transport to less-equipped facilities, necessitating interfacility transfers. Both scenarios contribute to delayed care and increased hospital and EMS resource utilization^7–9^. Given that stroke patients can lose up to 120 million neurons every hour^10^ and interfacility transfer times can average nearly 3 h^11^, accurate and timely prehospital data is critical for improving stroke patient triage and transport.

Prehospital data have the potential to enhance early clinical decision-making in EMS^12–16^. EMS personnel routinely collect a wide range of patient data, including demographics, vital signs, transit times, qualitative notes, and geospatial health determinants^17^. Some of these data elements, such as dispatch codes and nature of call, originate from the dispatch center and serve as early signals for EMS and, potentially, AI systems. The growing availability of prehospital data, coupled with advancements in interoperability between EMS and Emergency Department (ED) systems^18–20^, offers new opportunities for developing data-driven tools that can support stroke detection and improve prehospital decision-making^21,22^.

This study hypothesizes that machine learning (ML)-based predictive models powered by prehospital data can address the limitations of current prehospital stroke assessment scales and enhance diagnostic accuracy for time-sensitive conditions such as stroke^17,23^. Focusing on an EMS region in the western suburbs of Chicago, it assesses the availability and reliability of prehospital data by comparing vital signs collected by EMS with corresponding ED records to evaluate data consistency. Additionally, it develops multiple ML models to identify stroke and assess its severity, evaluating their potential to surpass existing prehospital assessment metrics.

## Materials and methods

### Study design and patient population

This retrospective study included 4,333 patients ≥ 18 years old transported by ambulance to Loyola University Medical Center (LUMC), a single university-affiliated Level I trauma center and comprehensive stroke center. All included patients were transported to this single emergency department by multiple fire-based EMS agencies operating in Illinois EMS Region 8, a designated region in Illinois’ regionalized EMS system that encompasses parts of the suburban Cook and DuPage counties in the greater Chicago area. The study included patients who had ≥ 1 prehospital vital sign measurement between January 1, 2015, and December 31, 2020.

Two additional subsets were examined using the same methods as the full cohort:

- **EMS-Suspected Stroke Cohort** – Encounters with a documented CPSS score (*n* = 1,252 encounters; 1,030 patients). A documented CPSS suggests that EMS suspected stroke at the time of assessment, making this group appropriate for comparing model performance directly with an established prehospital screening tool in the same clinical context.
- **Stroke and Mimic Cohort** – Encounters in which stroke was either confirmed by hospital diagnosis or plausibly considered by EMS. This group included all confirmed stroke encounters, all encounters in the EMS-Suspected Stroke Cohort, and all encounters where the dispatch “nature of call” suggested a potentially stroke-related presentation (e.g., Convulsions, Fall(s), Headache, Stroke/CVA, Syncope/Unconscious, Dizziness, Seizures) (*n* = 2,573 encounters; 1,873 patients). By limiting non-stroke cases to those with presentations plausibly consistent with stroke, this group reflects the real-world challenge of distinguishing strokes from stroke mimics and avoids inflating performance estimates that can occur when obvious non-stroke cases are included.

This study was reviewed and approved by the Loyola University Chicago Institutional Review Board under exemption category 45 CFR 46.104(d)(4)(ii-iii) for secondary research. All methods were carried out in accordance with relevant guidelines and regulations. The requirement for informed consent and patient authorization was waived by the Loyola University Chicago Institutional Review Board, as the study involved retrospective analysis of existing data and met criteria for minimal risk, impracticability of consent, and HIPAA privacy rule waiver.

### Study variables

The electronic Patient Care Report (ePCR) from a single institution’s ZOLL ePCR SQL server, storing EMS-treated patient data, was analyzed^24^. This system, compliant with the Illinois National EMS Information System (NEMSIS) version 3.4, standardizes EMS documentation to ensure consistent data collection and reporting^25^. Prehospital EMS records and ED data were linked at the patient-encounter level using available demographic and temporal fields, including patient name, date of birth, pickup address, and date of EMS dispatch and hospital arrival. This linkage was performed prior to the current study and did not rely on a shared unique identifier or HL7-based data exchange. Only encounters with a verified EMS-ED match were included.

Variable selection was limited to structured ePCR fields that were (1) routinely documented in our EMS region and (2) available for the majority of encounters. The following variables were extracted:

- **Demographics**: age, gender, race, ethnicity, weight, and body mass index (BMI).
- **Vital signs**: heart rate (HR), systolic (SBP) and diastolic blood pressure (DBP), respiratory rate (RR), oxygen saturation (SpO₂), temperature, blood glucose, pulse strength, respiration effort, and Glasgow Coma Scale (GCS) scores. Measurements outside the physiological ranges were considered missing values. The details of considered physiological ranges are provided in **Supplementary Table ****S1**.
- **Cincinnati Prehospital Stroke Scale (CPSS).** Null/Not Applicable/Not Known CPSS results were considered as normal results to assess EMS stroke recognition. Non-conclusive CPSS results were considered abnormal.
- **Transit time**: time from initial prehospital measurement to the initial ED measurement.
- **Pickup and drop-off locations**: geospatial data on transport routes.
- **Dispatch Information**: call type, call priority, transport priority, and nature of call. The ‘nature of call’ variable captures the dispatcher’s initial coded impression of the reason for the 9-1-1 call based on information provided by the caller. These categorical labels (e.g., “Stroke/CVA”, “Chest Pain”, “Breathing Problems”) are assigned before EMS arrival and are derived from structured dispatch software or manual dispatcher entry. A “Stroke/CVA” label reflects dispatch-level suspicion of stroke, while other labels may represent non-stroke or other presentations.
- **Patient Pickup Location**: The national Area Deprivation Index (ADI) was also assigned to each trip based on the pickup location. The ADI is a measure of socioeconomic disadvantage by neighborhood census block group, where higher values indicate increased disadvantage^26,27^.

The first available prehospital measurements taken at the scene before ambulance transport were used, as they represent the earliest opportunity for EMS intervention. If there were discrepancies between EMS-recorded arrival times and ED admission times, the earliest recorded time was used, following national stroke care abstraction guidelines^28^.

**Fig. 1:**
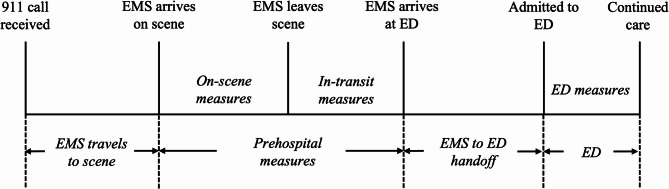
Flowchart of collection of prehospital and emergency department measurements.

Electronic health record (EHR) data from the ED were also used to extract patient demographics, comorbidities, and clinical measurements. Race/ethnicity was categorized into “Non-Hispanic White” (NHW), “Non-Hispanic Black” (NHB), “Non-Hispanic Other” (NHO) including Alaskan Native, American Indian, Asian, Multiracial, Native Hawaiian, and Other Race, and “Hispanic.” Initial ED vital signs, blood glucose, and GCS were used if recorded within four hours of ED admission. Stroke subtype diagnoses were derived from hospital discharge ICD-10 codes at hospital discharge: I60 for subarachnoid hemorrhage (SAH), I61 for intracerebral hemorrhage (ICH), and I63 for cerebral infarction. Severe strokes were defined using ICD-9 and Current Procedural Terminology (CPT) codes to identify patients with strokes who also had ventilation assistance and management (ICD-9: 96.70, 96.71, 96.72; CPT: 94002, 94003, 94004) and/or an intensive care unit admission (CPT: 99291, 99292). Prehospital and ED measurement timelines are illustrated in Fig. 1.

For encounters with documented National Institutes of Health Stroke Scale (NIHSS) scores in the ED, the first available NIHSS score was used as an additional clinical benchmark. We dichotomized NIHSS at ≥ 15 to approximate severe stroke, consistent with prior CSC triage criteria. For non-severe stroke, NIHSS 1–14 was used. NIHSS-based classification was compared descriptively to ICD/CPT-based labels and to model predictions. This allowed us to evaluate agreement between a gold-standard clinical severity assessment and our administrative/severity coding definitions.

### Statistical analysis to compare EHR and EMS

Descriptive statistics were used to summarize patient demographics. Pairwise independent t-tests compared clinical features between stroke and non-stroke patients. Spearman’s correlation coefficients (ρ), with 95% confidence intervals (CI) derived from Fisher’s z-transformation, assessed associations between prehospital and ED vital sign measurements. Scatter plots with regression lines illustrated these relationships. To evaluate linear associations while accounting for multiple observations per patient, multivariable Generalized Estimating Equation (GEE) models with a compound-symmetry correlation structure were used. Analyses adjusting for transit time are reported in the Supplementary Materials. Sensitivity analyses were conducted in stroke-confirmed subsets using similar models.

### Data preprocessing for machine learning

Two binary classification tasks were defined for model development and evaluation: (1) Stroke vs. non-stroke, and (2) Severe stroke vs. others. Variables with > 30% missing data were excluded, and missing values in continuous variables were imputed using scikit-learn’s IterativeImputer^29^. The dataset was split into stratified training (70%) and hold out testing (30%) subsets, followed by one-hot encoding for categorical variables. The same preprocessing steps were applied to both secondary datasets.

### Model training and evaluation

Three machine learning models were trained for each classification task: Random Forest (RF), XGBoost (XGB), and Sequential Neural Network (SNN). Hyperparameter tuning was conducted using Optuna, employing stratified 5-fold cross-validation to optimize AUC within a manually defined hyperparameter space^30^. To address class imbalance, class weights were adjusted by assigning higher weights to the minority class.

Machine learning models were also trained and evaluated on the subset of trips with valid CPSS values following the same methodology. For the expanded Stroke and Mimic subset, only RF and XGB were trained due to prior results showing little performance advantage for the SNN.

Model performance was evaluated using ROC-AUC and precision-recall AUC (PR AUC), with 95% confidence intervals estimated via nonparametric bootstrapping. For each model, 5,000 bootstrap resamples were drawn with replacement from the test set. In each resample, AUC and PR AUC were computed. The 95% CI bounds were calculated as the 2.5th and 97.5th percentiles of the resulting distribution of AUC values. Two-sided p-values < 0.05 were considered statistically significant.

To compare model performance to existing prehospital stroke screening tools such as CPSS^31^ and Vision, Aphasia, Neglect (VAN)^32^ without relying on a single threshold, we generated sensitivity-specificity tradeoff curves by plotting these metrics across classification thresholds from 0 to 1 in increments of 0.01. For each threshold, binary predictions were computed from model probabilities and the corresponding sensitivity and specificity values were calculated. In addition, we identified an ‘optimal’ operating threshold for each top-performing model using Youden’s J^33–35^, and reported the associated sensitivity, specificity, positive and negative likelihood ratios (LR + and LR-), and PPV at that threshold. These analyses were performed for the top-performing model for each classification task (stroke and severe stroke).

### Model interpretation and calibration

Feature importance was analyzed using SHAP values^36^ to assess predictor contributions. RF and XGB models were calibrated using polynomial non-decreasing calibration, adapted for imbalanced classification^37,38^. Calibration performance was evaluated with the average adaptive calibration error at 20%^38^, which averages calibration error across the highest probability quantiles to better assess high-risk (minority) predictions. SNN models were calibrated using attended temperature scaling^39,40^. All statistical analyses were performed using Python version 3.12.

### Code availability

The code used for data preprocessing, model training, evaluation, and visualization is available on GitHub (https://github.com/msabanluc/stroketriage-paper) and archived on Zenodo (10.5281/zenodo.17727869). All analyses were conducted in Python 3.12 using open-source libraries. No proprietary or identifiable data are included in the repository to protect privacy. Instructions for reproducing the analyses are provided in the repository’s README file.

## Results

### Patient characteristics

Among 4,333 patients age ≥ 18 years old with ≥ 1 prehospital vital sign measurement, the median age was 63 [IQR 50–76], 47.7% were men, median BMI was 27.8 kg/m^2^ [IQR 23.8–33.5], and race/ethnicity was 39.0% NHW, 54.3% NHB, 1.8% NHO, and 4.1% Hispanic. Table 1 provides patient characteristics among all prehospital encounters to the ED. Most patients (66.5%) had only one EMS encounter during the study period, 27.0% had 2–4 encounters, and 6.6% had ≥ 5 encounters.

**Table 1:** Characteristics of study population by patient and by encounter.

Demographics	All patients (*N* = 4333)	All encounters (*N* = 8221)
Age, median [Q1, Q3]	63 [50, 76]	64 [52, 78]
Gender, N (%)		
Female	2,268 (52.3%)	4,482 (55.7%)
Male	2,065 (47.7%)	3,639 (44.3%)
Race/ethnicity, N (%)		
Non-Hispanic White	1,690 (39.0%)	2,975 (36.2%)
Non-Hispanic Black	2,352 (54.3%)	4,815 (58.6%)
Non-Hispanic Other	79 (1.8%)	126 (1.5%)
Hispanic	175 (4.0%)	261 (3.2%)
Height (in), median [Q1, Q3]	66 [63, 70]	66 [63, 70]
Weight (lb), median [Q1, Q3]	176 [145, 214]	172 [141, 210]
BMI (kg/m^2^), median [Q1, Q3]	27.8 [23.8, 33.5]	27.4 [23.2, 33.1]
Stroke, N (%)		
Yes	157 (3.6%)	161 (2.0%)
No	4,176 (96.4%)	8,060 (98.0%)
Type of stroke, N (%)		
Cerebral infarction	126 (80.3%)	129 (80.1%)
Subarachnoid hemorrhage	6 (3.8%)	6 (3.7%)
Intracerebral hemorrhage	25 (15.9%)	26 (16.1%)

BMI, body mass index; Q1, first quartile; Q3, third quartile. Race/ethnicity was missing for 37 patients (44 visits), height for 2595 patients (4925 visits), weight for 1778 patients (3327 visits), and BMI for 2,644 patients (5021 visits).

Across 8,221 prehospital encounters to the ED, 161 (2.0%) were confirmed strokes, representing 157 (3.6%) unique patients. Most of these patients experienced a single stroke-related encounter. Stroke subtypes included 129 (80.1%) cerebral infarctions, 26 (16.1%) ICH, and 6 (3.7%) SAH. Among confirmed strokes, 103 (64%) were classified as severe. Comparisons between stroke and non-stroke cases are detailed in Supplementary Table S2.

Two secondary subsets were analyzed in addition to the full dataset. The CPSS subset included 1,252 encounters (1,030 patients), with 80 confirmed stroke encounters (6.4%). The expanded “Stroke and Mimic” subset included 2,573 encounters (1873 patients), with 161 confirmed stroke encounters (6.3%).

### Prehospital and ED vital signs

Distributions and ranges of vital signs recorded by EMS and ED are summarized in Table 2. Most prehospital vital signs (> 98%) were recorded at the scene, with < 2% measured during transport. The availability of prehospital and ED vital signs was as follows: HR, BP, RR, oxygen saturation, and GCS were recorded in > 88% of prehospital encounters. Blood glucose was available in approximately 50% of prehospital encounters and 74% of ED encounters. Temperature was recorded in < 7% of prehospital cases but was more frequently documented in the ED (> 93% of cases). While measurements exceeding physiological limits were excluded, they accounted for < 1% of prehospital data.

**Table 2:** Comparison of initial prehospital and emergency department measures among 8221 encounters (4333 patients).

	Initial prehospital measures			ED measures		
	*N*	Median [Q1, Q3]	Range	*N*	Median [Q1, Q3]	Range
Heart rate (bpm)	7,689	90 [78, 104]	4–228	7,933	86 [74, 100]	20–230
Systolic blood pressure	7,533	144 [128, 166]	36–300	7,935	139 [122, 157]	42–298
Diastolic blood pressure	7,276	82 [74, 92]	7–194	7,934	78 [67, 90]	13–202
Respiratory rate	7,724	18 [16, 18]	1–130	7,922	18 [16, 20]	2–64
SpO_2_ (%)	7,367	98 [96, 98]	8–100	7,904	98 [96, 100]	3–100
Temperature (°F)	590	98.1 [97.3, 98.8]	89.1–105.1	7,698	98.2 [97.8, 98.6]	48.2–105.7
Glucose (mg/dL)	4,292	125 [102, 166]	7–650	6,109	119 [100, 154]	14−1165
Glasgow Coma Scale Score	8,173	15 [15, 15]	3–15	6,478	15 [15, 15]	3–15

Q1, first quartile; Q3, third quartile; SpO_2_, oxygen saturation.

### Correlation between prehospital and ED measurements

**Fig. 2:**
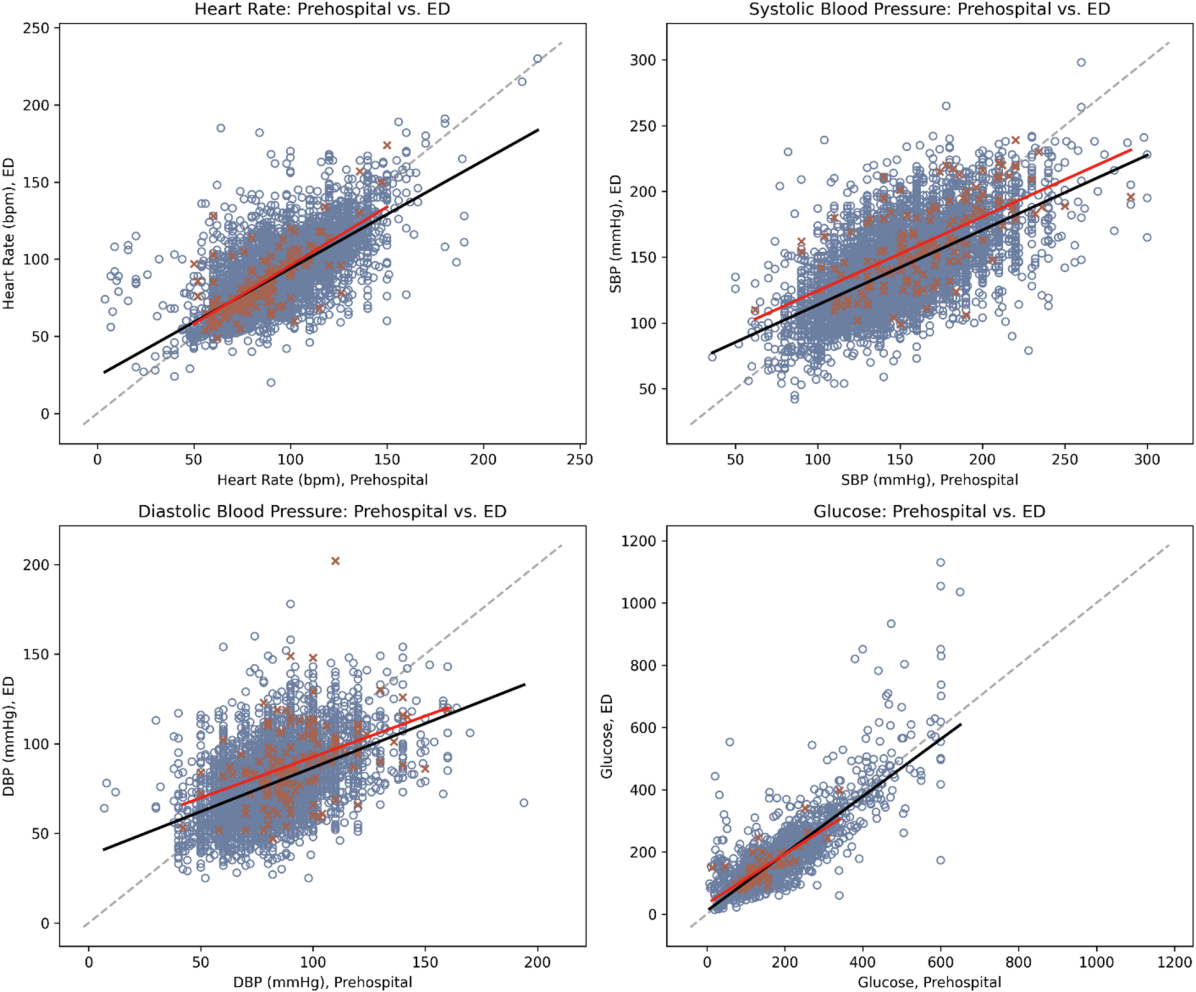
Scatterplots of prehospital vs. emergency department measurements with trendlines. The orange crosses (x) and blue circles (o) represent paired prehospital and ED values within four hours of ED arrival for stroke, and non-stroke patients, respectively. Solid red line represents encounters with strokes; solid black line represents encounters without strokes; dotted grey line represents 1:1 reference line.

Significant, strong positive correlations were observed for blood glucose (ρ = 0.73; 95% CI: 0.72, 0.75), HR (ρ = 0.72; 95% CI: 0.71, 0.73), and SBP (ρ = 0.62; 95% CI: 0.60, 0.63). In addition, moderate correlations were found for GCS (ρ = 0.52; 95% CI: 0.50, 0.53), DBP (ρ = 0.48; 95% CI: 0.45, 0.49), and temperature (ρ = 0.43; 95% CI: 0.36, 0.50), as shown in Supplementary Table S3. Weaker but significant correlations were observed for SpO₂ (ρ = 0.30; 95% CI: 0.28, 0.32) and RR (ρ = 0.21; 95% CI: 0.19, 0.23). Among confirmed stroke patients, these correlations remained consistent, except for RR, which showed no significant association (*p* = 0.74). Figure 2 illustrates these relationships. Additional analyses on the effect of transit time on ED measurements are provided in the Supplementary Tables S4-S5.

To address concerns regarding accuracy of ICD-10-based class definitions, we examined NIHSS scores for the 182 encounters with documented ED NIHSS values. NIHSS was recorded only after ED arrival and is not a prehospital screening tool. However, it does provide a clinical indicator of neurological deficit severity. Among encounters with NIHSS available, nearly all confirmed stroke cases had an NIHSS ≥ 1 (93.4%), while many non-stroke cases also had similar scores (specificity 19.8%). For severe stroke, NIHSS showed low sensitivity (19.2%) but high specificity (94.6%).

### Machine learning model performance

**Fig. 3:**
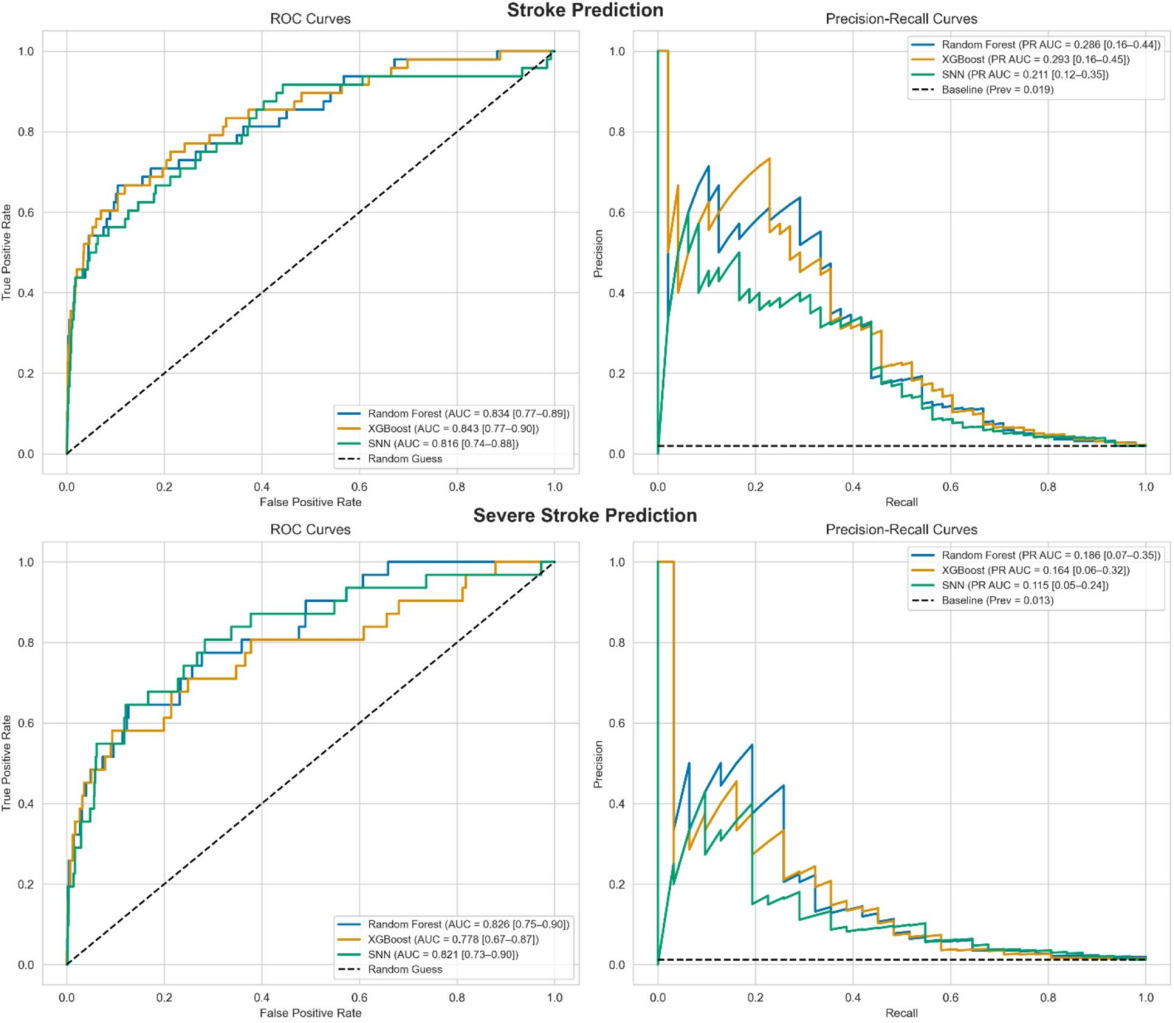
ROC and precision-recall curves comparing random forest, XGBoost, and sequential neural network models for stroke (top) and severe stroke (bottom) prediction. Dashed lines indicate chance (ROC) and baseline precision (PR).

Figure 3 shows the classification performance of each model for both stroke and severe stroke prediction. For stroke, all models showed similar ROC-AUC, with XGBoost achieving the highest (0.843 [95% CI: 0.77–0.90]), followed by Random Forest (0.834 [95% CI: 0.77–0.89]) and SNN (0.816 [95% CI: 0.74–0.88]). Precision-recall curves showed low PR-AUC values across models (XGB: 0.293, RF: 0.286, SNN: 0.211), which is consistent with the low stroke prevalence (2.0%).

Performance for severe stroke detection was generally lower. ROC-AUC values were again comparable across models (RF: 0.826 [95% CI: 0.75–0.90], XGB: 0.778 [95% CI: 0.67, 0.87], SNN: 0.821 [95% CI: 0.73–0.90]), with RF achieving the highest PR-AUC (0.186 [95% CI: 0.07–0.35]).

To allow direct comparison with existing tools, we present sensitivity, specificity, and PPV for the best-performing model in each task at a fixed probability threshold, determined by maximizing Youden’s J. Table 3 summarizes performance across decision thresholds (shaded rows mark the Youden-J maximum), and the resulting confusion matrices are shown in Table 4. Supplementary Fig. S1 plots sensitivity and specificity versus threshold for the full cohort and the EMS-suspected subgroup, with dashed/dotted lines indicating CPSS (data and literature) and VAN benchmarks. At these operating points, the XGBoost stroke model achieved 66.7% sensitivity, 88.0% specificity (LR + = 5.56; LR- = 0.38), and a PPV of 9.9%. The Random Forest severe stroke model achieved 64.5% sensitivity, 86.8% specificity (LR + = 4.89; LR- = 0.41), and a PPV of 5.9%.

**Table 3:** Threshold-based performance of the best models for stroke (XGBoost) and severe stroke (random forest) detection across three cohorts.

Source	Stroke				Severe stroke			
	Threshold	Sensitivity	Specificity	PPV	Threshold	Sensitivity	Specificity	PPV
Full cohort (*n* = 8221)								
ML model	0.005	0.979	0.275	0.026	0.005	0.806	0.625	0.027
	0.010	0.771	0.714	0.051	**0.010**	**0.645**	**0.868**	**0.059**
	0.015	0.688	0.812	0.068	0.015	0.516	0.917	0.074
	**0.025**	**0.667**	**0.880**	**0.099**	0.025	0.484	0.948	0.106
	0.100	0.458	0.977	0.282	0.100	0.323	0.976	0.145
EMS^*^	–	0.323	0.982	0.263	–	–	–	–
EMS-suspected stroke cohort (*n* = 1252)								
ML model	0.075	1.000	0.000	0.064	0.005	1.000	0.235	0.045
	**0.080**	**0.750**	**0.835**	**0.237**	**0.025**	**0.923**	**0.707**	**0.102**
	0.085	0.542	0.934	0.361	0.030	0.692	0.790	0.106
	0.100	0.458	0.940	0.344	0.100	0.385	0.945	0.200
	0.150	0.083	1.000	1.000	0.200	0.231	0.986	0.375
CPSS^‡^	–	0.650	0.875	0.263	–	–	–	–
CPSS (literature)^¶^	–	0.811	0.517	–	–	–	–	–
VAN (literature)^¶^	–	–	–	–	–	0.810	0.380	–
Stroke and mimic cohort (*n* = 2573)								
ML model	0.050	0.896	0.503	0.107	0.015	0.806	0.653	0.089
	0.055	0.812	0.624	0.125	0.020	0.774	0.692	0.095
	0.065	0.750	0.704	0.144	**0.030**	**0.742**	**0.760**	**0.114**
	**0.075**	**0.688**	**0.801**	**0.186**	0.040	0.677	0.794	0.121
	0.100	0.458	0.903	0.239	0.100	0.452	0.900	0.159

Performance is reported as Sensitivity, Specificity, and Positive Predictive Value (PPV) at selected decision thresholds. Cohorts are shown in bold: Full cohort (*n* = 8,221); EMS-Suspected Stroke Cohort (*n* = 1,252); Stroke and Mimic Cohort (*n* = 2,573). Shaded rows mark the rows that maximize Youden’s J within that cohort/outcome. ^*^EMS for stroke uses CPSS positivity in our dataset as a proxy for EMS suspicion, assuming undocumented CPSS represents a lack of EMS stroke suspicion; a severe-stroke analogue was not available. ^‡^CPSS (Data) is the performance of CPSS when documented. ^¶^CPSS (Literature) and VAN (Literature) are published benchmarks.

**Table 4:** Confusion matrices for the best models—stroke (XGBoost) and severe stroke (random forest)—across three cohorts.

	Positive: all stroke		Positive: all severe stroke	
	Predicted negative	Predicted positive	Predicted negative	Predicted positive
Full cohort (*n* = 8221)^*^				
True negative	2,127	291	2,114	321
True positive	16	32	11	20
EMS-suspected stroke cohort (*n* = 1252)^†^				
True negative	293	58	256	106
True positive	6	18	1	12
Stroke and mimic cohort (*n* = 2573)^‡^				
True negative	595	129	563	178
True positive	17	31	8	23

Results shown are for the held-out test set (30% of each cohort).

*Full cohort: Stroke – XGB t = 0.025; Severe – RF t = 0.010. ^†^EMS-Suspected cohort: Stroke – XGB t = 0.080; Severe – RF t = 0.025. ^**‡**^Stroke-related cohort: Stroke – XGB t = 0.075; Severe – RF t = 0.030.

To contextualize these results, we evaluated the standalone performance of dispatch-coded stroke suspicion using the ‘nature of call’ variable. Among the 8221 prehospital encounters, dispatcher-labeled stroke calls identified 73 of the 161 true strokes (sensitivity = 45.3%) and correctly excluded 7930 of 8060 non-stroke encounters (specificity = 98.4%). The positive predictive value was 36.0%, indicating that many dispatch-suspected strokes were false positives. In comparison, EMS stroke recognition had 32.3% sensitivity, 98.2% specificity, and 26.3% PPV across all 8,221 encounters (Supplementary Tables S6-S7).

### Model performance in EMS-suspected stroke cases

To compare with prior work restricted to EMS-suspected stroke encounters, we retrained models on the 1,252 cases with documented CPSS results. For stroke, the XGB model achieved the best performance with a ROC-AUC of 0.814 [95% CI: 0.70–0.92] and PR-AUC of 0.362 [95% CI: 0.18–0.54]. For severe stroke classification, RF had the best performance with a ROC-AUC of 0.858 [95% CI: 0.75–0.94] and PR-AUC of 0.322 [0.08–0.57]. Other models had comparable performance, with different trade-offs in sensitivity and specificity (Supplementary Fig. S2). Table 3 reports metrics across thresholds and Table 4 provides confusion matrices. At the thresholds that maximize Youden’s J, the XGBoost stroke model achieved a sensitivity of 75.0%, specificity of 83.5% (LR + = 4.55; LR- = 0.30), and a PPV of 23.7%, while the Random Forest severe stroke model achieved a sensitivity of 92.3%, specificity of 70.7% (LR + = 3.15; LR- = 0.11), and a PPV of 10.2%. For reference, CPSS within these 1,252 encounters achieved 65.0% sensitivity, 87.5% specificity (LR + = 5.2; LR- = 0.4), and a PPV of 26.3%.

### Model performance in stroke and mimic cohort encounters

We evaluated RF and XGB models on the expanded “Stroke and Mimic” subset. For stroke prediction, the XGB model achieved a ROC-AUC of 0.800 [0.73–0.87] and PR-AUC of 0.330 [0.20–0.46]. For severe stroke, the RF model achieved a ROC-AUC of 0.811 [0.75–0.88] and PR-AUC of 0.198 [0.09–0.33]. Table 3 highlights model performance across various thresholds and we report confusion matrices at representative thresholds (Table 4). XGB achieved a sensitivity of 68.8%, specificity of 80.1% (LR + = 3.46; LR- = 0.39), and a PPV of 18.6% on stroke prediction and RF achieved a sensitivity of 74.2%, specificity of 76.0% (LR + = 3.09; LR- = 0.34), and PPV of 11.4% on severe stroke prediction.

### Model interpretation and calibration

**Fig. 4:**
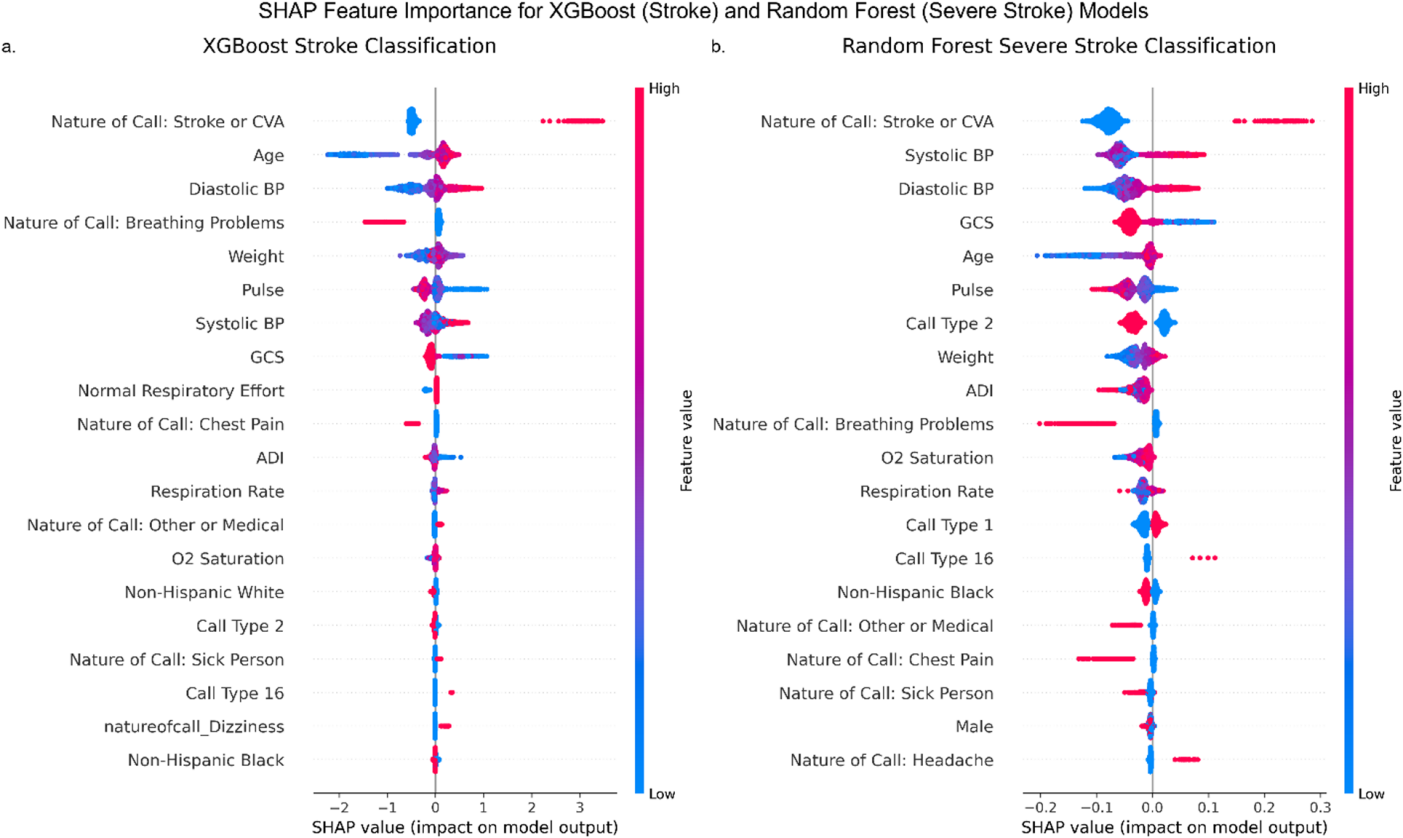
SHAP summaries of feature importance for the best-performing models in each binary classification task. (**a**) XGBoost for stroke classification and (**b**) Random Forest for severe stroke classification. Features are ranked by their impact on positive class predictions (stroke or severe stroke). Positive SHAP values indicate a higher likelihood of the outcome. Feature values are colored from low (blue) to high (red).

To improve interpretability, Fig. 4 presents SHAP value plots highlighting influential features in XGB (stroke) and RF (severe stroke) models. For both outcomes, key predictors included systolic and diastolic BP, GCS, age, pulse, weight, and stroke-relevant nature of call entries. This highlights the importance of physiological and dispatch data in early stroke recognition. Supplementary Fig. S3 illustrates model calibration before and after post-hoc adjustment. Both XGB and RF models were miscalibrated initially, with RF in particular overestimating probabilities for stroke. Calibration improved after adjustment, especially in the top 20% of predicted probabilities, as quantified by the Avg-ACE@20% score (XGB: 0.0390; RF: 0.0398). These improvements increase the reliability of probability estimates, which is essential for informed triage decisions in EMS.

## Discussion

This study evaluated the availability and reliability of prehospital EMS data from 8,221 adult encounters and demonstrated the utility of ML models for early identification of stroke and severe stroke. We benchmarked model performance against standard EMS stroke screening tools, including CPSS and VAN, and assessed model calibration, interpretability, and performance across three cohorts: the full population, a subset of patients with documented CPSS results (EMS-suspected stroke), and an expanded “Stroke and Mimic” subset that included all stroke encounters, all encounters with a valid CPSS, and encounters where the dispatch “nature of call” suggested potentially stroke-related presentation. Across these analyses, our findings highlight both the promise and limitations of using routinely collected structured EMS data for triage support.

### Prehospital data collection and EMS operational challenges

Prehospital vitals were highly available (> 88% for most features), with the exception of glucose (50%) and temperature (7%). While measurements generally aligned with ED values, certain vitals, such as respiratory rate, often repeated across encounters, suggesting inaccuracies likely due to manual entry or measurement challenges during transport. Similarly, temperature recordings were infrequent, limiting their predictive utility. ZOLL monitors can auto-populate vitals in systems like emsCharts^41^, which improves completeness^42^, but adoption and integration vary across agencies. These findings highlight the importance of improving device accuracy, documentation workflows, and EMS training to maximize utility of prehospital data for early triage.

Over 98% of prehospital measurements were documented at the scene, with < 2% in transit, reflecting focus on direct care during transport. However, discrepancies between EMS-reported arrival times and ED admission times (median difference: 2 min [Q1, Q3: 1, 6]) introduce potential errors in assessing EMS travel efficiency and operational performance. These inconsistencies impact broader clinical and logistical assessments and highlight the need for standardized documentation protocols to enhance prehospital data utility, decision-making, and patient outcomes^43–45^.

Prehospital data have demonstrated utility in improving in-hospital emergency care^17^. EMS personnel routinely relay prehospital information such as last known well, symptom onset, and initial vitals to ED personnel during handoff. These data are often crucial for timely stroke treatment decisions. However, while EMS narratives and assessments are verbally communicated, they are not always structured, integrated, or readily available in the electronic medical record in a way that facilitates automated decision support. Several systems have been developed to improve EMS-ED data integration, including electronic ambulance records and digital handover protocols^46,47^, but workflow integration is limited by technical and organizational barriers such as information access, cross-vendor interoperability, and patient matching challenges^48^. Documentation quality can also vary within EMS regions by software, device integration, staffing, and training. Addressing these barriers could improve the completeness and timeliness of prehospital data available for ED decision-making and ML-based triage tools, ultimately reducing treatment delays, eliminating redundant testing, and streamlining acute care pathways. This is particularly relevant for time-sensitive conditions such as stroke, where early intervention minimizes permanent neurological damage^10,49^.

### AI applications for stroke detection in the EMS setting

AI is increasingly utilized in emergency medicine to enhance stroke detection, particularly through imaging-based and EHR-driven models^50,51^, informing treatment decisions^22,52–54^, and predicting various treatment and functional outcomes^55–60^. However, relatively few AI-based studies focus on stroke prediction in the prehospital setting. Our work aligns with and expands on previous AI-driven prehospital stroke detection studies. Dispatch-level work by Wenstrup et al. showed that deep learning on call center audio improves early stroke recognition over human dispatchers^61^. Our study complements this earliest stage by focusing on the on scene phase by integrating dispatcher-coded fields such as ‘nature of call’ with structured, patient-level data collected by EMTs. While our dispatch data are less rich audio than audio, our models benefit from more detailed physiological data collected on scene.

Uchida et al. and Hayashi et al. used machine learning models to classify stroke subtypes (e.g., SAH, ICH, LVO) based on prehospital data^12,13^. Yoshida et al. developed ML models to predict which stroke patients require surgical intervention, such as mechanical thrombectomy, using EMS data^14^. Our work expands on these studies by addressing the critical question of where to transport stroke patients based on the resources available at different facilities. In the United States, the prehospital workflow is very different.

Stroke centers are rigorously evaluated and awarded certification by national accrediting and certifying bodies^62^. Certified stroke centers operate within a stroke system of care model where each level of stroke center has progressively increasing availability and complexity of care, treatment, and services provided to meet the needs of their patient populations^63^. For example, comprehensive stroke centers (CSCs), the highest level of certification, are required to provide 24/7 neurointerventional and neurosurgical services for all complex stroke patients subtypes. Additional considerations must also be made for the variability of prehospital stroke triage and transport that is subject to individual state routing legislation. Effective prehospital triage not only requires identifying stroke patients but also determining their severity to ensure they are transported to the most appropriate facility in accordance with applicable law and regulation. This makes it particularly challenging to develop scalable models within and across countries with varying stroke center classifications and healthcare systems. By defining severe stroke patients as those requiring ventilation or intensive care, our study includes patients in need of complex care and/or intervention that is provided at CSCs.

### Limitations of prehospital stroke screening tools

Current prehospital stroke recognition relies on traditional stroke screening assessments. The CPSS, which is derived from the National Institutes of Health Stroke Scale (NIHSS) and assesses facial movement, arm movement, and speech, is widely used across EMS systems^31^. In a national study of EMS data, CPSS was the most frequently documented stroke scale, being applied in about 61% of cases with documented stroke scale use^64^. However, this method suffers from highly variable performance and does not account for stroke severity or atypical symptoms. A recent review reported a prehospital CPSS sensitivity and specificity of 81.1% and 51.7%, respectively^65^. In our dataset, EMS identified only 32.3% of strokes across all 8,221 encounters, despite a specificity of 98.2% and PPV of 26.3%. Among the 1252 cases with documented CPSS results, the tool achieved 65.0% sensitivity, 87.5% specificity, and 26.3% PPV (Supplementary Tables S6-S7). However, only 80 of 161 confirmed stroke cases (50%) had a recorded CPSS assessment, suggesting that over half of stroke cases were not suspected by EMS at the time of transport. This finding highlights a key limitation in traditional screening tools. CPSS is only applied when stroke is suspected, meaning that patients with subtle or atypical presentations may be missed. Prior ML models for prehospital stroke detection have typically been trained only on EMS-suspected stroke cases, excluding patients who were later diagnosed in the hospital but initially missed by EMS. This can bias model development towards patients with classic stroke presentations and limit generalizability. Recent dispatch-level work by Wenstrup et al. similarly included all hospital-confirmed strokes (excluding SAH). Our approach builds on this by providing a comprehensive evaluation of ML’s potential to enhance stroke detection using on scene EMS-collected physiologic and clinical data, enabling evaluation of AI’s potential across a broader segment of the prehospital workflow.

A key limitation in assessing prehospital stroke recognition is the incomplete availability of EMS impressions in the dataset. In the absence of such a datapoint, CPSS documentation was used as a proxy for EMS stroke suspicion. While this aligns with paramedic feedback, which indicates that CPSS is typically performed when stroke is suspected and often omitted when suspicion is low, it cannot completely distinguish between true absence of suspicion and incomplete documentation. Missing CPSS values may reflect low suspicion, undocumented assessments, or technical/workflow-related gaps in documentation. This uncertainty may bias EMS stroke recognition estimates. Future work should incorporate structured impressions or NLP-extracted impressions from EMS narratives to obtain a more accurate measurement of prehospital stroke recognition.

### ML model performance and feature interpretation

Our ML models achieved competitive performance across three distinct cohorts despite relying solely on routinely documented, structured prehospital variables with sufficient completeness. Key variables such as last known well time, symptom onset, and glucose levels^66^ were often missing. Unstructured but potentially valuable data (e.g., ECG data^67^, EMS narratives^68^ were not included.

In the **full cohort**, the top-performing models achieved higher sensitivity than both dispatch and EMS recognition, identifying more true strokes, but with lower PPV due to the low baseline prevalence. This setting represents the broadest EMS case mix and the least challenging classification task, as most non-stroke encounters are obvious non-strokes.

In the **EMS-Suspected Stroke Cohort**, direct comparisons to existing tools were possible. Here, our ML models for stroke and severe stroke detection outperformed CPSS and literature-reported VAN in sensitivity while maintaining competitive specificity, but the restriction to documented CPSS cases excluded many true strokes and stroke-like presentations, limiting generalizability.

The **Stroke and Mimic Cohort** was developed to better reflect the true real-world challenge of distinguishing strokes from plausible mimics. As expected, model performance in this subset was more modest, reflecting the increased diagnostic complexity, but still showed balanced sensitivity and specificity.

Across all cohorts, trade-offs between sensitivity, specificity, and PPV were evident. The low PPV observed in the full cohort is consistent with its low prevalence, and while this reduces alert efficiency, higher sensitivity may be clinically valuable in time-critical conditions like stroke, provided model outputs are used to prompt expedited assessment rather than as definitive diagnoses. Likelihood ratios further contextualize model performance. LR + for stroke detection ranged from about 3 to 6, indicating that a positive model prediction makes true stroke 3–6 times more likely than in non-stroke cases. LR- ranging from about 0.3 to 0.4 shows that a negative prediction reduces the probability of stroke. Overall, these likelihood ratios present modest, but clinically relevant shifts in model output diagnoses.

Our severe stroke model addresses a different target population than VAN, which was developed for large vessel occlusion (LVO) detection and was not implemented in our EMS region during the study period^32^. Our comparison with VAN relies on literature-reported performance rather than direct analysis. A prospective prehospital study in Texas reported VAN’s sensitivity, specificity, and PPV as 81%, 38%, and 29%, respectively^69^. While not directly comparable, our models aim to identify a broader range of patients requiring CSC-level care, including neurointensive care admissions, suggesting a complementary role rather than a replacement for existing LVO-focused tools. Other EMS systems use scales such as the Los Angeles Motor Scale (LAMS), Rapid Arterial oCclusion Evaluation (RACE) scale, and FAST-ED, which were not documented in our dataset. Instead, our models relied on routinely collected physiologic and clinical variables.

Feature attribution analysis using SHAP identified physiologically and clinically meaningful predictors for both stroke and severe stroke, including systolic and diastolic BP, GCS score, pulse, respiratory rate, weight, and stroke-related dispatch codes (Fig. 4). While “nature of call” was one of the most influential features, our analysis suggests that this dispatch suspicion alone is insufficient for accurate stroke detection. In our dataset, dispatcher suspicion of stroke had a sensitivity of only 45.3%, a specificity of 98.4%, and a PPV of 36.0%. These limitations are important to consider when interpreting model performance. Although nature of call provides useful early information, the model’s ability to identify strokes not caught by dispatch demonstrates the value of incorporating EMT-collected data. Regardless, future work should quantify the independent contribution of ambulance-collected variables and evaluate the potential of incorporating richer dispatch data such as free-text notes, software responses, or audio transcripts to improve early recognition and allow for more nuanced modeling.

These results support the clinical validity of our models, showing that predictions are based on physiologically meaningful signals. Feature interpretation is important in AI-driven clinical decision making, but model probabilities are just as important. While post-hoc adjustment showed improved calibration, indicating better alignment between predicted and observed probabilities, calibrated probabilities were often low even for true stroke/severe stroke cases, which reflects the class imbalance and challenge of clinical interpretation of raw probabilities. For example, a 30% predicted probability may appear low to paramedics, but could correspond to a high-risk case relative to baseline.

### Future directions and clinical implications

Performance patterns across the three cohorts provide insight into both the potential and the limitations of ML-based prehospital stroke detection. In the full cohort, the models demonstrate the feasibility of applying ML to large, heterogeneous EMS datasets and highlight the capacity to detect stroke cases missed by EMS. This cohort reflects the true prevalence of stroke in the general EMS population but presents a relatively less complex classification task. In the EMS-Suspected Stroke Cohort, the models provide a direct comparison with established prehospital screening tools, showing improved sensitivity while maintaining acceptable specificity. This provides insight into potential real-world performance gains if ML support were added to existing workflows. However, this subset excludes a substantial proportion of true strokes. The Stroke and Mimic Cohort addresses a more clinically realistic diagnostic challenge by approximating the scenario EMS personnel face when differentiating stroke from plausible mimics. This cohort narrows the task to patients where stroke was at least plausible at some point in the prehospital process. The balanced performance metrics suggest potential clinical value in augmenting EMS decision making for this higher risk group.

These findings reinforce our overarching goal of using ML models to supplement existing EMS screening tools and clinical judgement. Utilizing structured, routinely collected data, these models could function as supplemental decision support by flagging high risk patients who might otherwise be missed during initial EMS evaluation.

Given the low prevalence of stroke, optimizing threshold strategies will be essential to balance false positives with the potential benefits of earlier detection. In the full cohort, this trade-off translates to a PPV of ~ 10%, or approximately 291 non-stroke alerts for every 32 true strokes detected (Table 4). This is expected when predicting rare events like stroke but could create practical challenges for field implementation. A high volume of stroke alerts may contribute to unnecessary overcrowding in the ED and affect EMT adherence unless outputs are clearly interpretable and context specific. Successful integration will require careful threshold selection and prospective workflow testing to ensure that alerts result in timely assessment without overburdening EMS personnel.

Likelihood ratios help further clarify the clinical impact of these models. LR + values around 3–6 indicate that while a positive prediction meaningfully increases the probability of stroke or severe stroke, they are insufficient to diagnose as a stand-alone tool. LR- values around 0.3–0.4 similarly reduce, but do not eliminate the probability of stroke. One exception is the severe stroke model within the EMS-Suspected Stroke Cohort, which had a LR- of about 0.11, which does show strong potential for ruling out patients already suspected of stroke. In practice, these likelihood ratios suggest that the model outputs would be most useful as flags that prompt further assessment rather than definitive diagnostic tools.

Future work should prioritize prospective, multi-site validation to assess generalizability across regions and EMS systems with differing protocols. Integration strategies into EMS workflows should be tested to ensure compatibility with existing processes and minimal disruption. Expanding the feature set to include unstructured data sources, such as EMS narratives and ECG traces, could enhance predictive accuracy and better capture clinically relevant information.

Efforts to address fairness are also essential. Although we explored fairness adjustments using Fairlearn^70^, extreme class imbalance limited the ability to improve equalized odds across demographic subgroups without substantial performance trade-offs. Future model development will need to prioritize strategies that balance fairness and performance, potentially through improved data collection or alternative modeling approaches.

Several additional limitations require consideration. Time related analyses may be affected by discrepancies between EMS arrival times and ED admission timestamps. The single region, single hospital nature of the dataset may limit external validity, emphasizing the need for larger, geographically diverse datasets. Hospital diagnosis relied on validated ICD-10 codes (I60, I61, I63), which demonstrate high positive predictive value in administrative datasets^71,72^. While diagnostic coding is not always accurate, it remains the most practical and widely used method for large-scale retrospective studies. To further assess the outcomes, we examined NIHSS scores where available. However, NIHSS was documented in only 2.1% of encounters, had low specificity for distinguishing stroke from mimics, and showed poor sensitivity for identifying our definition of severe stroke. As noted earlier, EMS stroke recognition may also be imperfectly captured, as CPSS documentation was used as a proxy for EMS suspicion.

Finally, successful implementation of ML-based support tools will require understanding operational realities, including EMS personnel engagement, acceptance, integration with existing protocols, and potential impacts on the workflow. Identifying practical barriers and facilitators early will be essential for translating these models into tools that enhance prehospital decision making for time sensitive conditions like stroke.

## Conclusions

This study demonstrates the feasibility of leveraging routinely collected prehospital EMS data, analyzed through a ML approach, for early stroke detection and triage. The high availability and strong correlation of prehospital measurements with ED values highlight their potential utility in clinical decision-making. At the same time, broader inconsistencies in EMS documentation highlight the need for improved standardization to ensure reliability for AI-driven decision support.

Our ML models exhibited promising predictive performance, with the ability to identify stroke cases missed by existing processes. These findings reinforce the potential role of ML as a supplemental tool in prehospital stroke triage to augment EMS clinical judgement. Future research should prioritize improving data consistency across EMS systems, validating models prospectively in diverse settings, and integrating multimodal data sources such as ECG readings and prehospital narratives. By advancing AI-driven prehospital triage, this work lays the groundwork for more efficient patient transport decisions, ensuring timely interventions and improved stroke outcomes.

## Supplementary Information

Below is the link to the electronic supplementary material.

Supplementary Material 1

## Data Availability

The datasets generated and/or analyzed during the current study are not publicly available due to IRB restrictions and institutional privacy policies but are available from the authors upon reasonable request and with permission of the Loyola University Chicago Institutional Review Board.

## Abbreviations

BMI: Body mass index
BP: Blood pressure
DBP: Diastolic blood pressure
GCS: Glasgow coma scale
SBP: Systolic blood pressure

## Code availability

Code availability is described in section 2.7.
