# Supplementary material for: Machine learning models powered by emergency medical services data enhance stroke triage in prehospital settings
Source: Sci Rep. 2026 Feb 3;16:7139. doi: 10.1038/s41598-026-37069-x (PMC12920803; doi:10.1038/s41598-026-37069-x)

**Supplementary Table S1.** Physiological ranges of prehospital and emergency department measurements

| Variable | Definition |
| --- | --- |
| Heart rate | Greater than 0 beats per minute (bpm) but less than or equal to 300 bpm |
| Systolic blood pressure | Greater than 0 mmHg but less than or equal to 300 mmHg |
| Diastolic blood pressure | Greater than 0 mmHg but less than or equal to 200 mmHg |
| Respiratory rate | Greater than 0 breaths per minute |
| SpO_2_ (%) | Greater than 0% but less than or equal to 100% |
| Temperature | Between 80.0^o^ F and 110^o^ F |
| Glucose | Greater than 0 mg/dL but less than or equal to 1,500 mg/dL |

**Supplementary Table S2**. Comparisons of stroke vs. non-stroke patients and encounters.

| Demographics | Stroke patients  (N=157) | Non-stroke patients  (N=4,176) | p-value | Stroke encounters  (N=161) | Non-stroke encounters  (N=8,060) | p-value |
| --- | --- | --- | --- | --- | --- | --- |
| Age | 72 [63, 81] | 62 [49, 76] | <0.01* | 72 [63, 81] | 64 [52, 78] | <0.01* |
| Gender, N (%) |  |  | 0.48 |  |  | 0.97 |
| Female | 87 (55.4%) | 2,181 (52.2%) |  | 89 (55.3%) | 4,493 (55.7%) |  |
| Male | 70 (44.6%) | 1,995 (47.8%) |  | 72 (44.7%) | 3,567 (44.3%) |  |
| Race/Ethnicity, N (%) |  |  | 0.42 |  |  | 0.02 |
| Non-Hispanic White | 60 (38.2%) | 1,630 (39.0%) |  | 61 (37.9%) | 2,914 (36.2%) |  |
| Non-Hispanic Black | 83 (52.9%) | 2,269 (54.3%) |  | 85 (52.8%) | 4,730 (58.7%) |  |
| Non-Hispanic Other | 5 (3.2%) | 74 (1.8%) |  | 6 (3.7%) | 120 (1.5%) |  |
| Hispanic | 6 (3.8%) | 169 (4.0%) |  | 6 (3.7%) | 255 (3.2%) |  |
| Height (in) | 66 [63, 69] | 66 [63, 70] | 0.24 | 66 [63, 69] | 66 [63, 70] | 0.50 |
| Weight (lb) | 185.6 [153.4, 228.4] | 175.0 [145.0, 212.0] | 0.01* | 185.6 [153.0, 228.4] | 171.3 [140.8, 209.0] | <0.01* |
| BMI (kg/m^2^) | 29.9 [25.6, 35.4] | 27.6 [23.7, 33.3] | <0.01* | 29.9 [25.6, 35.5] | 27.4 [23.1, 33.0] | <0.01* |
| Prehospital measurements | 1 [1, 2] | 1 [1, 2] | 0.23 | - | - | - |

BMI, body mass index; Q1, first quartile; Q3, third quartile. Race/ethnicity was missing for 34 patients (44 visits), height for 2,438 patients (4,764 visits), weight for 1,621 patients (3,166 visits), and BMI for 2,487 patients (4,860 visits). Data shown as median (IQR) for age, vitals, and prehospital measurement count.

**Supplementary Table S3.** Spearman’s correlation coefficients between initial prehospital vital signs and emergency department vital signs.

|  | Prehospital vs. ED measures  (All encounters) | | | Prehospital vs. ED measures  (Any Stroke) | | |
| --- | --- | --- | --- | --- | --- | --- |
|  | **N** | $\boldsymbol{\rho}$ **(95% CI)** | **p-value** | **N** | $\boldsymbol{\rho}$ **(95% CI)** | **p-value** |
| Heart rate | 7,623 | 0.72 (0.71, 0.73) | <0.01 | 155 | 0.68 (0.59, 0.76) | <0.01 |
| Systolic blood pressure | 7,480 | 0.62 (0.60, 0.63) | <0.01 | 154 | 0.65 (0.54, 0.73) | <0.01 |
| Diastolic blood pressure | 7,226 | 0.47 (0.45, 0.49) | <0.01 | 151 | 0.46 (0.33, 0.58) | <0.01 |
| Respiratory rate | 7,652 | 0.21 (0.18, 0.23) | <0.01 | 156 | 0.03 (-0.13, 0.18) | 0.74 |
| Oxygen saturation | 7,297 | 0.30 (0.28, 0.32) | <0.01 | 152 | 0.23 (0.08, 0.38) | <0.01 |
| Temperature | 582 | 0.43 (0.36, 0.50) | <0.01 | 16 | 0.57 (0.11, 0.83) | 0.02 |
| Glucose | 3,618 | 0.73 (0.72, 0.75) | <0.01 | 82 | 0.72 (0.60, 0.81) | <0.01 |
| Glasgow Coma Scale | 6,450 | 0.52 (0.50, 0.53) | <0.01 | 118 | 0.66 (0.55, 0.75) | <0.01 |

ED, emergency department.

**Supplementary Table S4.** Emergency department measurements regressed on prehospital measurements for full cohort, adjusting for time between measurements (per 10-minute increase in transit time). Among the full cohort, the slope estimates are less than one for all measurements, indicating the magnitudes of all ED measurements were, on average, lower than their corresponding prehospital measurements. For example, per one beat-per-minute increase in prehospital HR, the ED HR was expected to only increase by 0.7 (95% CI: 0.7, 0.7; p<0.01). When controlling for the prehospital measurement, increasing transit time was significantly associated with decreasing ED measurements for all variables except for SpO₂ and GCS. For example, each 10-minute interval in transit time was associated with a 0.6 mmHg (95% CI: 0.4, 0.9; p<0.01) lower ED SBP compared to the prehospital SBP.

|  | N | Intercept estimate  (95% CI) | p-value | Change in ED estimate per increase in prehospital measurement  (95% CI) | p-value | Change in ED estimate per 10-minute increase in transit time  (95% CI) | p-value |
| --- | --- | --- | --- | --- | --- | --- | --- |
| Heart rate | 7,593 | 25.0 (22.7, 27.3) | <0.01 | 0.7 (0.7, 0.7) | <0.01 | -0.3 (-0.5, -0.1) | <0.01 |
| Systolic blood pressure | 7,453 | 58.6 (55.6, 61.7) | <0.01 | 0.6 (0.6, 0.6) | <0.01 | -0.6 (-0.9, -0.4) | <0.01 |
| Diastolic blood pressure | 7,202 | 39.6 (37.3, 41.9) | <0.01 | 0.5 (0.5, 0.5) | <0.01 | -0.6 (-0.8, -0.5) | <0.01 |
| Respiratory rate | 7,624 | 15.6 (14.5, 16.8) | <0.01 | 0.2 (0.1, 0.3) | <0.01 | -0.1 (-0.1, -0.1) | <0.01 |
| Oxygen saturation | 7,267 | 82.8 (79.1, 86.5) | <0.01 | 0.2 (0.1, 0.2) | <0.01 | 0.0 (0.0, 0.0) | 0.74 |
| Temperature | 578 | 44.2 (35.3, 53.1) | <0.01 | 0.6 (0.5, 0.6) | <0.01 | -0.0 (-0.1, 0.0) | 0.02 |
| Glucose | 3,572 | 20.0 (9.7, 30.3) | <0.01 | 0.9 (0.8, 1.0) | <0.01 | -1.4 (-1.8, -1.0) | <0.01 |
| Glasgow Coma Scale | 6,391 | 8.2 (7.4, 9.1) | <0.01 | 0.4 (0.4, 0.5) | <0.01 | 0.0 (0.0, 0.0) | 0.11 |

CI, confidence interval; ED, emergency department.

**Supplementary Table S5.** Emergency department measurements regressed on prehospital measurements for the stroke cohort, adjusting for time between measurements (per 10-minute increase in transit time). Compared to Table S4, the prehospital and ED measurements were no longer significantly associated with each other for RR (p=0.42), SpO_2_ (p=0.15), and temperature (p=0.07). Similarly, transit time was no longer significantly associated with the ED measurements, except for temperature, which showed a 0.1 ^o^F (95% CI: 0.0, 0.1) lower ED temperature compared to the respective prehospital measurements.

|  | N | Intercept estimate  (95% CI) | p-value | Change in ED estimate per increase in prehospital measurement  (95% CI) | p-value | Change in ED estimate per 10-minute increase in transit time  (95% CI) | p-value |
| --- | --- | --- | --- | --- | --- | --- | --- |
| Heart rate | 154 | 23.4 (5.3, 41.4) | 0.01 | 0.7 (0.6, 0.9) | <0.01 | -0.9 (-2.2, 0.4) | 0.19 |
| Systolic blood pressure | 153 | 64.7 (45.3, 84.2) | <0.01 | 0.6 (0.5, 0.7) | <0.01 | 0.9 (-1.8, 3.6) | 0.52 |
| Diastolic blood pressure | 151 | 48.4 (33.8, 63.0) | <0.01 | 0.5 (0.3, 0.6) | <0.01 | -0.5 (-2.0, 1.0) | 0.54 |
| Respiratory rate | 155 | 16.3 (9.9, 22.8) | <0.01 | 0.2 (-0.2, 0.5) | 0.42 | 0.0 (-0.2, 0.3) | 0.86 |
| Oxygen saturation | 151 | 86.8 (71.2, 102.3) | <0.01 | 0.1 (0.0, 0.3) | 0.15 | -0.0 (-0.2, 0.2) | 0.78 |
| Temperature | 16 | 39.7 (-23.2, 102.6) | 0.22 | 0.6 (0.0, 1.2) | 0.07 | -0.1 (-0.1, 0.0) | <0.01 |
| Glucose | 81 | 43.7 (10.3, 77.0) | 0.01 | 0.8 (0.6, 1.0) | <0.01 | -1.7 (-3.8, 0.3) | 0.09 |
| Glasgow Coma Scale | 116 | 3.6 (-1.2, 8.4) | 0.14 | 0.7 (0.4, 1.1) | <0.01 | -0.0 (-0.2, 0.1) | 0.43 |

**Supplementary Table S6.** Comparison of prehospital stroke scale assessment results with true labels.

|  | Stroke Scale Screening Assessment | | | |  |
| --- | --- | --- | --- | --- | --- |
|  | Normal | Abnormal | Non-Conclusive | Not Applicable/Known/Available | NaN |
| Non-Stroke | 1,026 | 81 | 65 | 159 | 6,729 |
| Non-Severe Stroke | 15 | 17 | 5 | 0 | 21 |
| Severe Stroke | 13 | 29 | 1 | 1 | 59 |

Categorizes stroke scale results across all 8,221 encounters, including cases where CPSS was not performed (NaN values).

**Supplementary Table S7.** Confusion matrices of EMS and CPSS performance in stroke identification.

| True Label | Predicted Non-Stroke  (All Encounters) | Predicted Stroke  (All Encounters) | Predicted Non-Stroke  (CPSS Subset) | Predicted Stroke  (CPSS Subset) |
| --- | --- | --- | --- | --- |
| True Non-Stroke | 7,914 | 146 | 1,026 | 146 |
| True Stroke | 109 | 52 | 28 | 52 |

‘All Encounters’ includes all patient transports (n=8,221), while the ‘CPSS Subset’ is limited to trips where CPSS was performed (n=1,252). In both, non-severe and severe strokes are grouped as ‘True Stroke’, and ‘Abnormal’ or ‘Non-Conclusive’ CPSS results are grouped as ‘Predicted Stroke’. For all encounters, ‘Predicted Non-Stroke’ includes ‘Normal’, ‘Not Applicable/Known/Available’, and missing CPSS values, while the missing values and ‘Not Applicable/Known/Available’ are excluded for the CPSS subset.

**Supplementary Figure S1.** Sensitivity-specificity tradeoff curves for ML-based classification of stroke and severe stroke using XGBoost (Stroke) and Random Forest (Severe Stroke). Panels (a) and (b) show model performance on all EMS encounters (n=8,221), while panels (c) and (d) are restricted to encounters where EMS suspected stroke (n=1,252). ML sensitivity and specificity curves are shown across probability thresholds. In panel (a), dashed lines represent estimated EMS benchmarks based on CPSS values from our dataset, assuming that missing values reflect no stroke suspicion. In contrast, panel (c) shows true CPSS performance within the suspected stroke subgroup using actual CPSS assessments from our data (dashed lines) and from literature (dotted lines). For severe stroke, panel (d) includes dotted lines representing literature-reported EMS benchmarks for VAN.

**
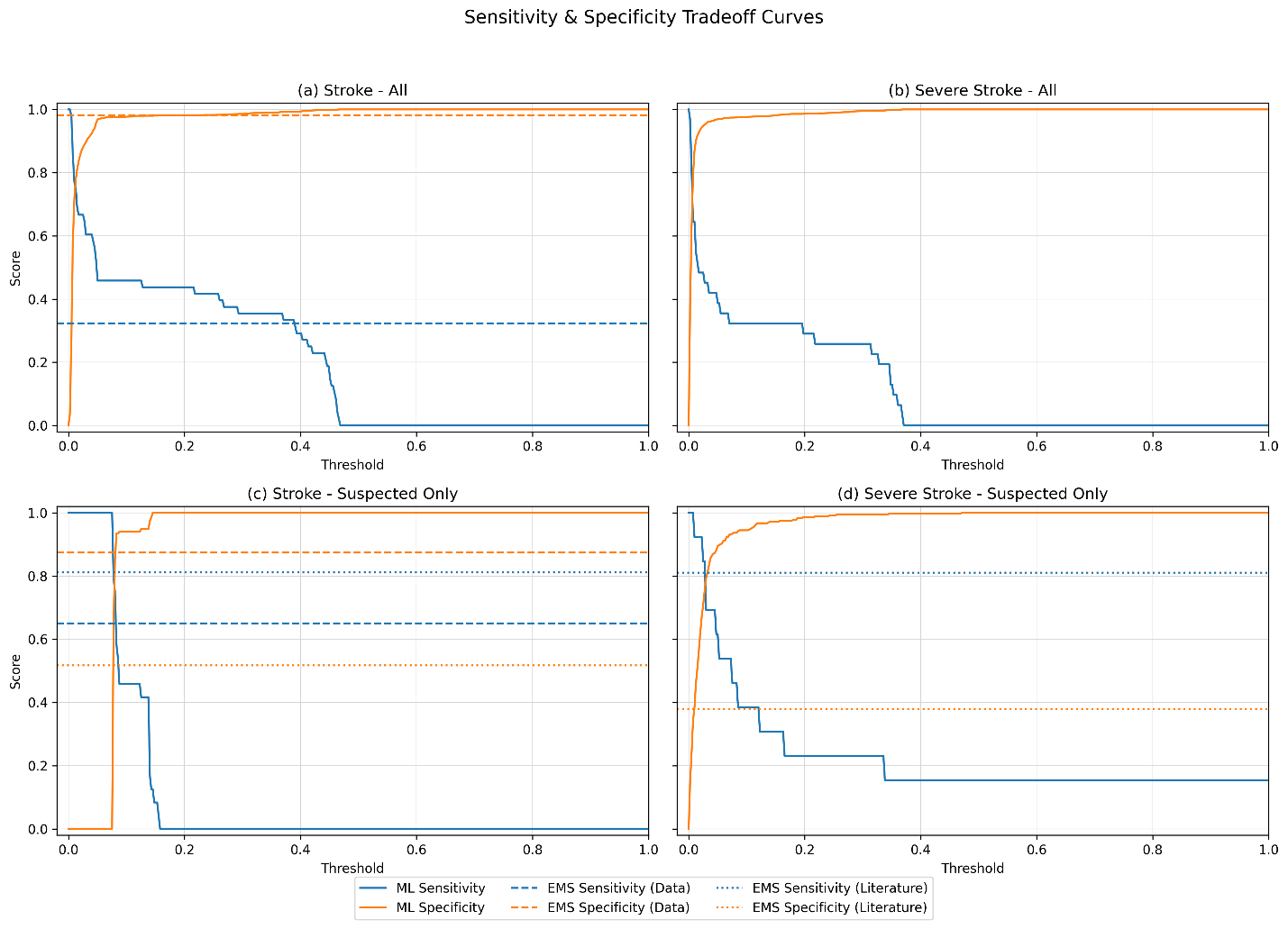
**

**Supplementary Figure S2.** ROC and precision-recall curves comparing random forest, XGBoost, and sequential neural network models for stroke (top) and severe stroke (bottom) prediction on the EMS stroke-suspected subset (n=1,252). Dashed lines indicate chance (ROC) and baseline precision (PR)


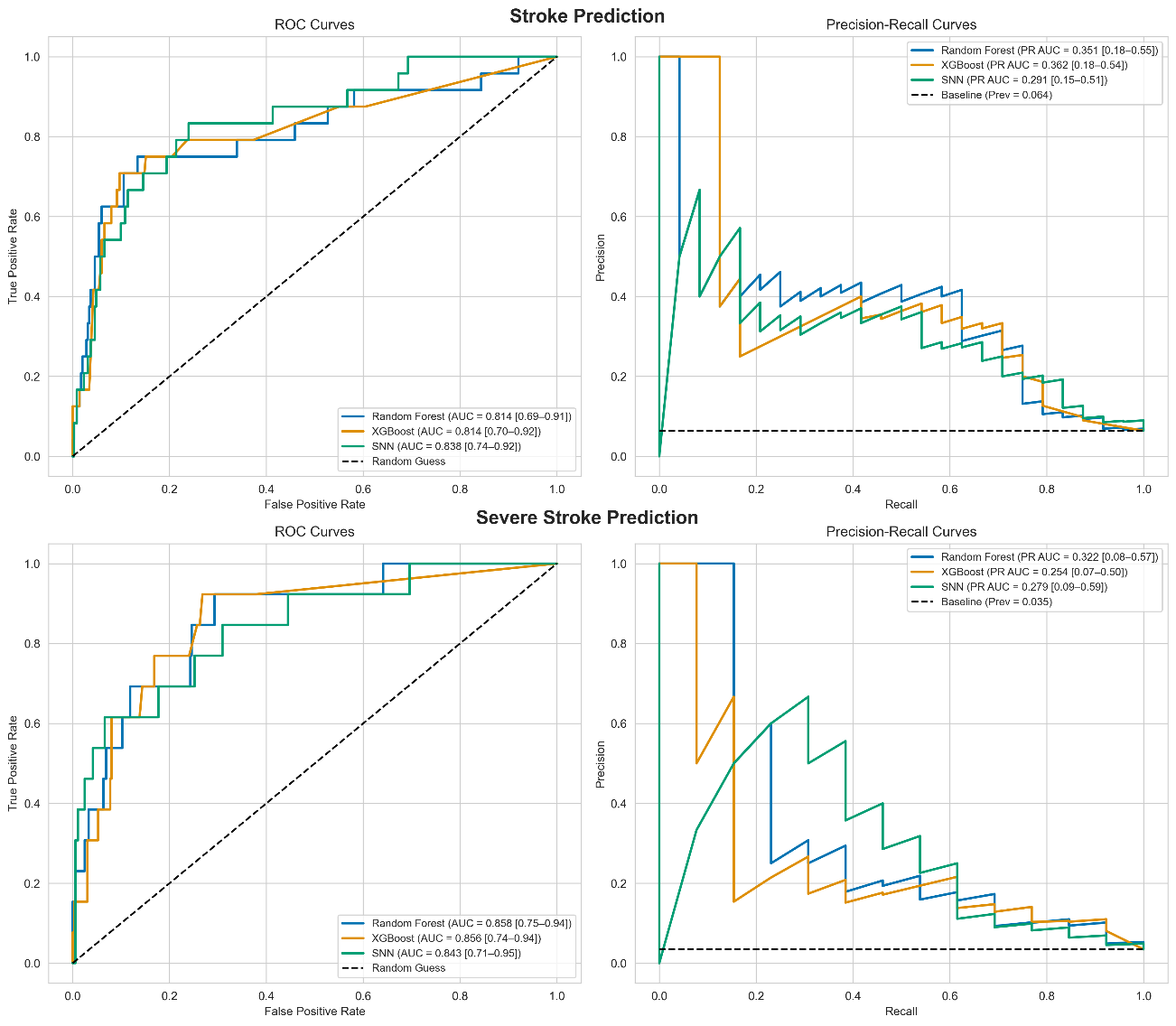


**Supplementary Figure S3.** Reliability diagrams comparing uncalibrated (left) and calibrated (right) XGBoost and Random Forest models for stroke and severe stroke classification, respectively. Solid lines represent model reliability, while the dashed line indicates perfect calibration. Avg-ACE@20% (Average Adaptive Calibration Error in the top 20% of predicted probabilities) quantifies miscalibration, with lower values indicating better calibration.


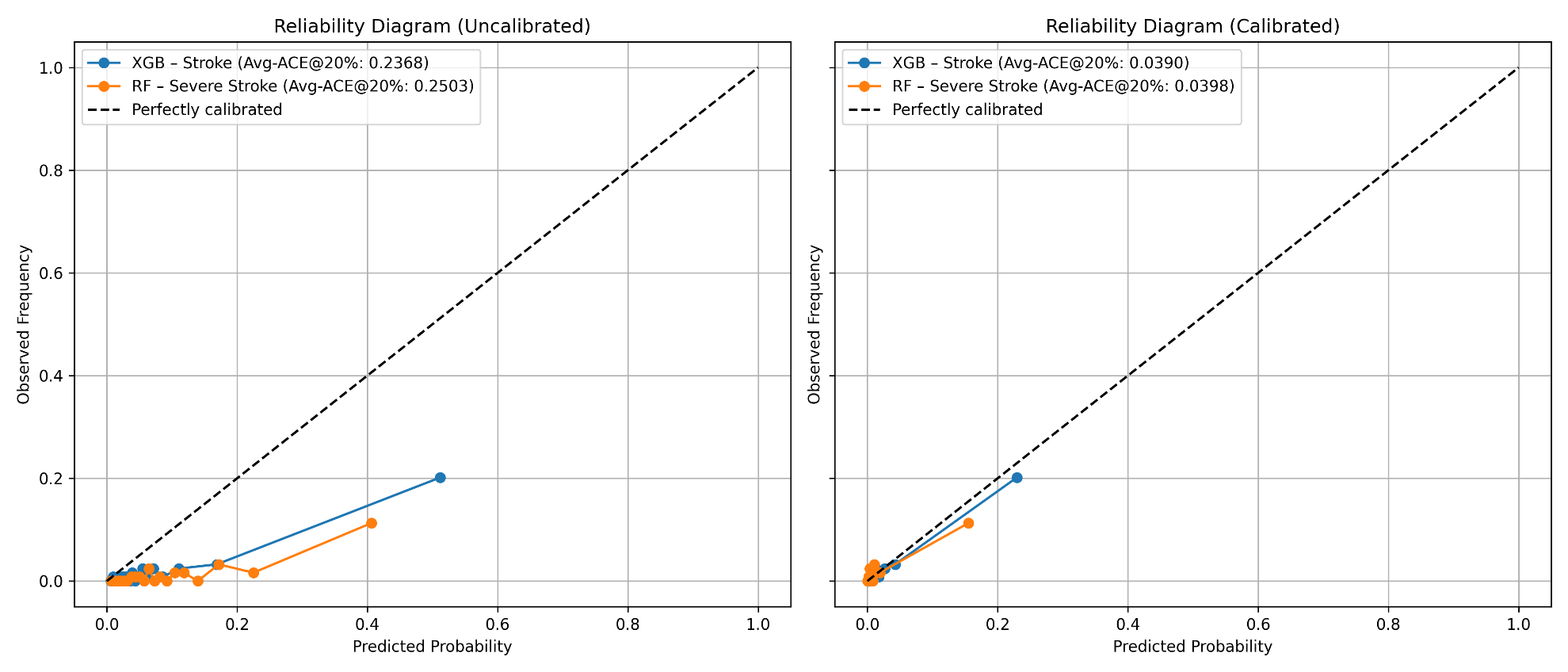

Supplement: Supplementary file 1 — Supplementary Material 1 [file 41598_2026_37069_MOESM1_ESM.docx]
